# Repair or replace ischemic mitral regurgitation during coronary artery bypass grafting? A meta-analysis

**DOI:** 10.1186/s13019-016-0536-6

**Published:** 2016-09-01

**Authors:** Yushu Wang, Xiuli Shi, Meiqin Wen, Yucheng Chen, Qing Zhang

**Affiliations:** Department of Cardiology, West China Hospital, Sichuan University, Chengdu, China

**Keywords:** Ischemic mitral regurgitation, Mitral valve repair, Mitral valve replacement, Coronary artery bypass grafting, Meta-analysis

## Abstract

**Background:**

No agreement has been reached for the best surgical treatment for patients with chronic ischemic mitral regurgitation (IMR) undergoing coronary artery bypass grafting (CABG). Our objective was to meta-analyze the clinical outcomes of repair and replacement.

**Methods:**

A computerized search was performed using Pubmed, Embase, Ovid medline and Cochrane Library. The search terms “ischemic or ischaemic” and “mitral valve” and “repair or replacement or annuloplasty” and “coronary artery bypass grafting” were entered as MeSH terms and keywords. The primary outcomes were operative mortality and late mortality. Secondary outcomes were 2+ or greater recurrence of mitral regurgitation and reoperation rate.

**Results:**

Eleven studies were eligible for the final meta-analysis. These studies included a total of 1750 patients, 60.4 % of whom received mitral valve repair. All patients underwent concomitant coronary artery bypass graft. No differences were found in operative mortality (summary odds ratio [OR] 0.65; 95 % confidence interval [CI] 0.43-1.00; *p* = 0.05), late mortality (summary hazard ratio [HR] 0.87; 95 % confidence interval [CI] 0.67-1.14; *p* = 0.31) and reoperation (summary odds ratio [OR] 1.47; 95 % confidence interval [CI] 0.90-2.38; *p* = 0.12). Regurgitation recurrence was lower in the replacement group (summary odds ratio [OR] 5.41; 95 % confidence interval [CI] 3.12-9.38; *p* < 0.001).

**Conclusion:**

In patients with chronic ischemic mitral regurgitation during CABG, mitral valve replacement is associated with lower recurrence of regurgitation. No differences were found regarding survival and reoperation rates.

## Background

Chronic ischemic mitral regurgitation (IMR) is a frequent and important complication after myocardial infarction. Its pathophysiologic mechanisms account for remodeling of segmental/global left ventricle (LV) inducing papillary muscle displacement and leaflet tethering [1]. The presence of IMR is independently associated with mortality and morbidity after myocardial infarction [2].

Given the severity of IMR, surgery performed for IMR ranges from coronary artery bypass grafting (CABG) alone to both CABG and mitral valve surgery [3, 4]. Two randomized trials indicated that repair was associated with a reduced prevalence of mitral regurgitation but did not show a clinically meaningful advantage of adding mitral valve repair to CABG [5, 6]. In addition, when compared with replacement, previous meta-analyses concluded that repair is associated with lower operative mortality but higher recurrence of regurgitation in patients with ischemic mitral regurgitation, with or without CABG [7, 8]. For patients with chronic IMR undergoing combined CABG, the best surgical treatment is still controversial. Some studies support replacement [9, 10], others support repair [11, 12], and others showed similar survival for the two procedures [13]. Current guidelines recommend mitral valve surgery for severe IMR, but do not demonstrate a specific type of procedure [14, 15]. Numerous non-randomized studies have been published comparing the clinical outcomes between MVP + CABG and MVR + CABG for IMR. However, there is still no systematic and quantitative assessment of accumulated literature on this topic. Meta-analysis is a powerful tool to provide meaningful comparison of short and long-term outcomes of these procedures. The present meta-analysis aimed to assess the clinical outcomes of patients who underwent mitral valve surgery and CABG for chronic IMR.

## Methods

### Search strategy

This meta-analysis was conducted according to the recommendations of the Meta-Analysis of Observational Studies in Epidemiology (MOOSE) [16]. A computerized search was performed using Pubmed, Embase, Ovid medline and Cochrane Library from their dates of inception to December 2015 without language restriction. The search terms “ischemic or ischaemic” and “mitral valve” and “repair or replacement or annuloplasty” and “coronary artery bypass grafting” were entered as MeSH terms and keywords. The language of publication was restricted to English. We also reviewed the full text and references lists of all relevant review articles in detail. YW and XS independently undertook the literature search, screening of titles and abstracts. Any disagreement was resolved by consensus.

### Study selection

Articles were included if there is a direct comparison of repair versus replacement and all patients with IMR had CABG. The exclusion criteria were applied to select the final articles for the meta-analysis: (1) ischemic etiology in only a subset of the patients with outcomes not specifically provided (2) nonischemic dilated cardiomyopathy (3) beating heart procedures (4) concomitant surgical ventricular restoration (5) preoperative hemodynamic instability (6) lack of annuloplasty in > 20 % of the patients in the repair group (7) acute IMR.

### Data extraction and quality assessment

All data were extracted independently by 2 investigators (Y.W., X.S.) according to the prespecified selection criteria, with disagreement resolved by consensus among all authors. The following data from each study were extracted: the last name of the first author, year of publication, study population, patients’ age and gender, comorbidities, cardiac function, severity of mitral regurgitation at baseline and follow-up period. Any disagreement was resolved by consensus.

Based on the extracted data, the quality of the included studies was evaluated using the nine-item Newcastle-Ottawa Quality scale [17], a widely used tool for the quality assessment of non-randomized trials. The high-quality study was defined as a study with ≥6 scores.

### Statistical analysis

The primary end points were operative mortality and late mortality (considered to be year after operation). Operative mortality was defined as death within 30 days after operation or in-hospital death. Secondary end points were MR recurrence 2+ or greater and reoperation at follow-up. The meta-analysis was performed using Review Manager (Revman, version 5.3 for windows, Oxford, England, Cochrane Collaboration) and Stata (version 11.0; StataCorp, College Station, TX). Hazard ratio (HR) with a 95 % confidence intervals (CIs), directly extracted from these included studies or indirectly calculated using the method of Tierney and colleagues [18] to assess the efficacy of the surgical intervention in each study. A summary of odds ratio (OR) and their corresponding 95 % CI were computed for each dichotomous outcome using either fixed-effects models or, in the presence of substantial heterogeneity (I^2^ > 50 %), random-effects models [19]. Statistical heterogeneity across studies was examined with Cochran’s Q test as well as the I^2^ statistics. Studies with an I^2^ statistics of <25 % were considered to have low heterogeneity, those with an I^2^ statistics of 25–50 % were considered to have moderate heterogeneity, and those with an I^2^ statistics of >50 % were considered to have a high degree of heterogeneity [20]. If there was high heterogeneity, the possible clinical and methodological factors for this were further explored. Potential sources of heterogeneity were investigated using sensitivity analyses and each study involved in the meta-analysis was excluded each time to reflect the influence of the individual data set on the pooled RRs.

Publication bias was assessed using the Egger regression asymmetry test [21] and Begg adjusted rank correlation test [22]; a *P* value of less than 0.05 was considered representative of statistically significant publication bias. Meta-analysis results are displayed in forest plots. A *p* value < 0.05 was considered statistically significant.

## Results

### Search results and study quality

**Fig. 1:**
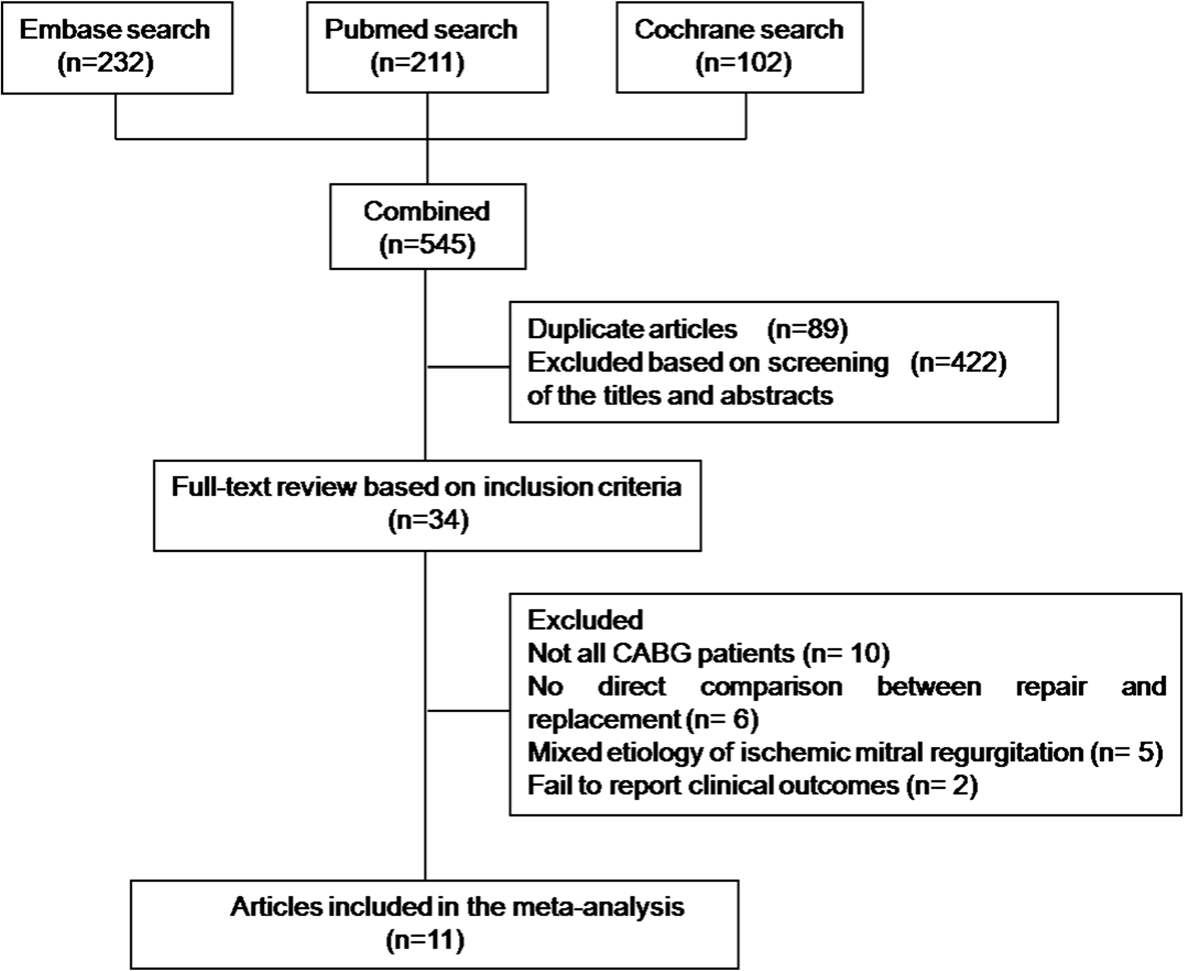
Flow chart of study selection

The literature search identified a total of 545 studies, which were published between 1965 and 2015. On the basis of title and abstracts, 34 articles were selected and reviewed in full. Eleven articles met the inclusion and exclusion criteria [9–13, 23–28] (Fig. 1). Of the included studies, there were ten retrospective observational studies [9–13, 23–27] and one prospective observational study [28]. All were nonrandomized studies. These studies included a total of 1807 patients, 1091 (60.4 %) of whom underwent repair and 716 (39.6 %) of whom underwent replacement. All patients had CABG. Patient characteristics and a summary of operative details are summarized in Tables 1 and 2, respectively. With the exception of the replacement patients being older in 2 of the studies, the two groups were similar in terms of hypertension (HTN), diabetes, atrial fibrillation (AF), left ventricular ejection fraction (LVEF) and the New York Heart Association (NYHA) class. Eight of the studies reported data on the type of prosthesis used for mitral valve replacement and preservation of the subvalvular apparatus. In half of the studies, the majority of patients received a bioprothesis valve. In addition, preservation of the subvalvular (either total or partial) apparatus were performed in the vast majority of mitral valve replacements.

**Table 1:** Key Features of Included Studies

Study	Subjects		Mean Age		Male (%)		HTN (%)		Diabetes (%)		AF (%)		NYHA III-IV (%)		Mean LVEF (%)		MR grade		Follow-up period
	MVP + CABG	MVR + CABG	MVP + CABG	MVR + CABG	MVP + CABG	MVR + CABG	MVP + CABG	MVR + CABG	MVP + CABG	MVR + CABG	MVP + CABG	MVR + CABG	MVP + CABG	MVR + CABG	MVP + CABG	MVR + CABG	MVP + CABG	MVR + CABG	
Lorusso et al.	244	244	66	66	73	69	41	41	36	35	12	13	NR	NR	35	35	2.8 ± 0.5	2.8 ± 0.5	46.5^b^mo
Lio et al.	98	28	65	70^d^	74	61	81	89	35	32	NR	NR	61	71	32	34	NR	NR	45^b^mo
Ljubacev et al.	34	41	NR	NR	NR	NR	85	80	32	56^d^	26	17	NR	NR	NR	NR	NR	NR	In-hospital
Roshanali et al.	26	31	57	57	83	77	NR	NR	NR	NR	NR	NR	NR	NR	38	40	3.6 ± 0.5	3.5 ± 0.5	40.2^a^ mo
Maltais et al.	302	85	70	70	68	63	71	68	34	26	NR	NR	85	91	34	34	NR	NR	4.2^a^yrs
Qiu et al.	112	106	71	72	64	56	72	75	30	32	28	26	53	49	35	35	NR	NR	48.1^a^mo
Micovic et al.	86	52	61^b^	62^b^	72	73	74	65	21	15	27	29	64	50	29	36	2.7 ± 0.6	2.5 ± 0.7	32^a^ mo
Bonacchi et al.	36	18	NR	NR	NR	NR	NR	NR	NR	NR	NR	NR	NR	NR	27	27	NR	NR	32^a^ mo
Silberman et al.	38	14	62	67^d^	74	93	50	57	45	57	NR	NR	49^cd^	32^cd^	<25 %		NR	NR	38^a^mo
Mantovani et al.	61	41	68	68	67	54	54	51	26	15	NR	NR	NR	NR	45	45	3.1 ± 0.8	3.3 ± 0.7	36.8^a^ mo
Reece et al.	54	56	67	69	41^d^	68^d^	NR	NR	22	21	NR	NR	NR	NR	44	40	NR	NR	In-hospital

^a^ = mean; ^b^ = median; ^c^ Percentage class IV; ^d^ *p* < 0.05 between MVr and MVR

*Abbreviations: AF* atrial fibrillation, *LVEF* left ventricular ejection fraction, *CABG* coronary artery bypass grafting, *MR* mitral regurgitation, *HTN* hypertension, *MVP* mitral valve repair, *MVR* mitral valve replacement, *NR* not reported

**Table 2:** Operative characteristics

	CPB time (min)		ACC time (min)		MVR prosthesis type		Subvalvular apparatus preservation			MVP partial/suture annuloplasty (%)	MVP ring annuloplasty (%)	MVP undersizing
	MVP	MVR	MVP	MVR	Mechanical %	Bioprothesis %	Anterior + Posterior (%)	Posterior (%)	None (%)			
Lorusso et al. [9]	145	145	94	94	47	53	48	24	43	0	100	27 (26 mm) 52 (28 mm) 13 (30 mm) 6 (32 mm) 1 (34 mm) 1 (36 mm)
Lio et al. [10]	156	180	107	132	36	64	100	0	0	0	100	37 % open ring 63 % closed ring 37 % rigid ring 63 % semi-rigid ring
Ljubacev et al. [24]	145	152	96	99	NR	NR	NR	NR	NR	NR	NR	NR
Roshanali et al. [28]	NR	NR	NR	NR	100	0	100	0	0	NR	NR	NR
Maltais et al. [13]	NR	NR	NR	NR	46	54	NR	NR	NR	8	92	42 (24–28 mm) 36 (30–34 mm)
Qiu et al. [26]	136	129	105	98	38	62	11	89	0	0	100	30 mm
Micovic et al. [11]	NR	NR	NR	NR	100	0	0	100	0	5	95	Median 28 mm (range, 26–34 mm)
Bonacchi et al. [12]	NR	NR	NR	NR	NR	NR	0	100	0	17	83	NR
Silberman et al. [23]	154	184	99	111	100	0	NR	NR	NR	0	100	26 ± 1.2 mm
Mantovani et al. [25]	179	173	131	122	76	24	0	100	0	0	100	Moderate
Reece et al. [27]	112	132	152	171	NR	NR	NR	NR	NR	0	100	28 mm males 26 mm females

*Abbreviations: MVP* mitral valve repair, *MVR* mitral valve replacement, *ACC time* aortic cross-clamping time, *CPB time*, cardiopulmonary bypass time, *NR* not reported

All the eleven trials were assessed by the Newcastle-Ottawa Scale for quality assessment risk evaluation of adequacy of selection, comparability, and outcomes assessment for individual trials (Table 3). All studies included in our meta-analysis were of high-quality (had ≥ 6 scores).

**Table 3:** Study quality assessment using the Newcastle-Ottawa Scale for nonrandomized studies

	Selection					Outcome			
First author, year of publication (reference)	Representativeness of exposed cohort	Selection of nonexposed cohort	Ascertainment of exposure	Outcome of interest absent at start of study	Comparability (Based on design and analysis)	Assessment of outcome	Follow-up long enough for outcomes to occur	Adequacy of follow-up	Total score
Lorusso et al.	1	1	1	1	0	1	1	1	7
Lio et al.	1	1	1	1	1	1	1	1	8
Ljubacev et al.	1	1	1	1	0	1	0	1	6
Roshanali et al.	1	1	1	1	2	1	1	1	9
Maltais et al.	1	1	1	1	2	1	1	1	9
Qiu et al.	1	1	1	1	2	1	1	1	9
Micovic et al.	1	1	1	1	2	1	1	1	9
Bonacchi et al.	1	1	1	1	2	1	1	1	9
Silberman et al.	1	1	1	1	1	1	1	1	8
Mantovani et al.	1	1	1	1	2	1	1	1	9
Reece et al.	1	1	1	1	1	1	0	1	7

### Peri-operative mortality

**Fig. 2:**
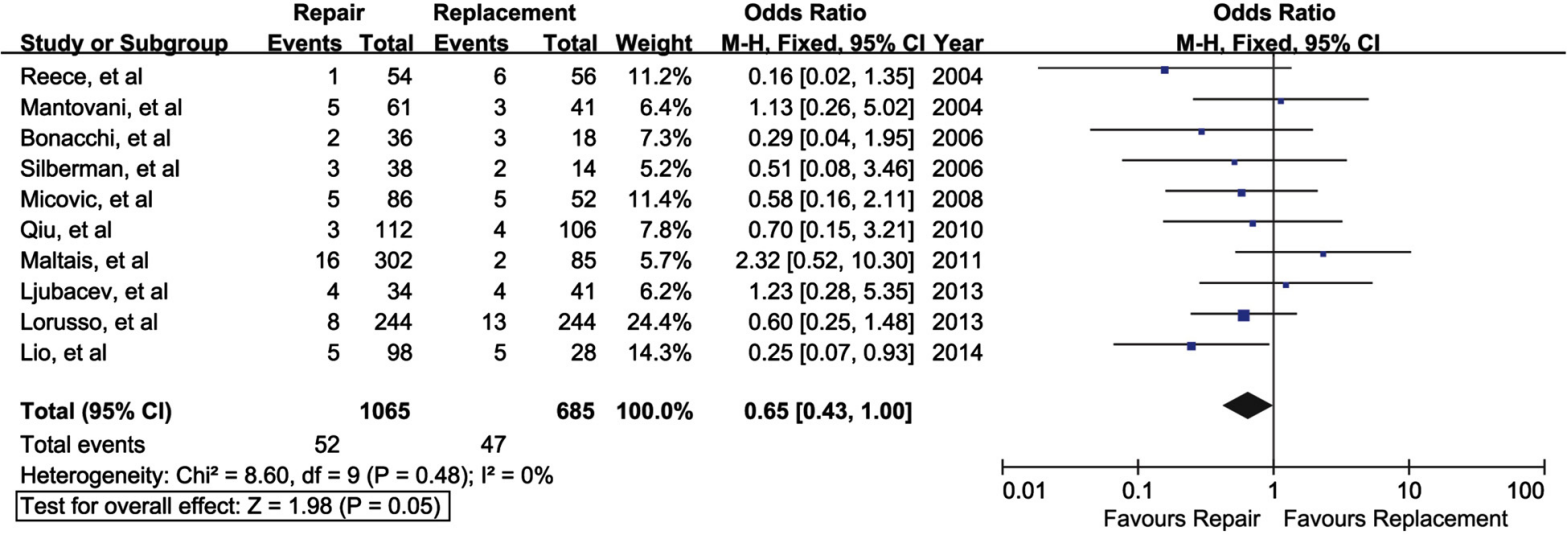
Mitral valve repair versus mitral valve replacement on peri-operative mortality

Ten observational studies involving a total of 1750 patients reported operative mortality. The odds ratios in the study ranged from 0.16 to 2.32 (Fig. 2). The summary odds ratio was 0.65 (95 % CI, 0.43-1.00), *P* = 0.05, indicating there was a reduced peri-operative mortality trend towards repair, but no statistical significance reached. In assessing potential heterogeneity across the studies, I^2^ = 0 %, and no publication bias was found either from the Egger’s test (*P* = 0.83) or the Begg’s test (*P* = 0.68).

### Late mortality

**Fig. 3:**
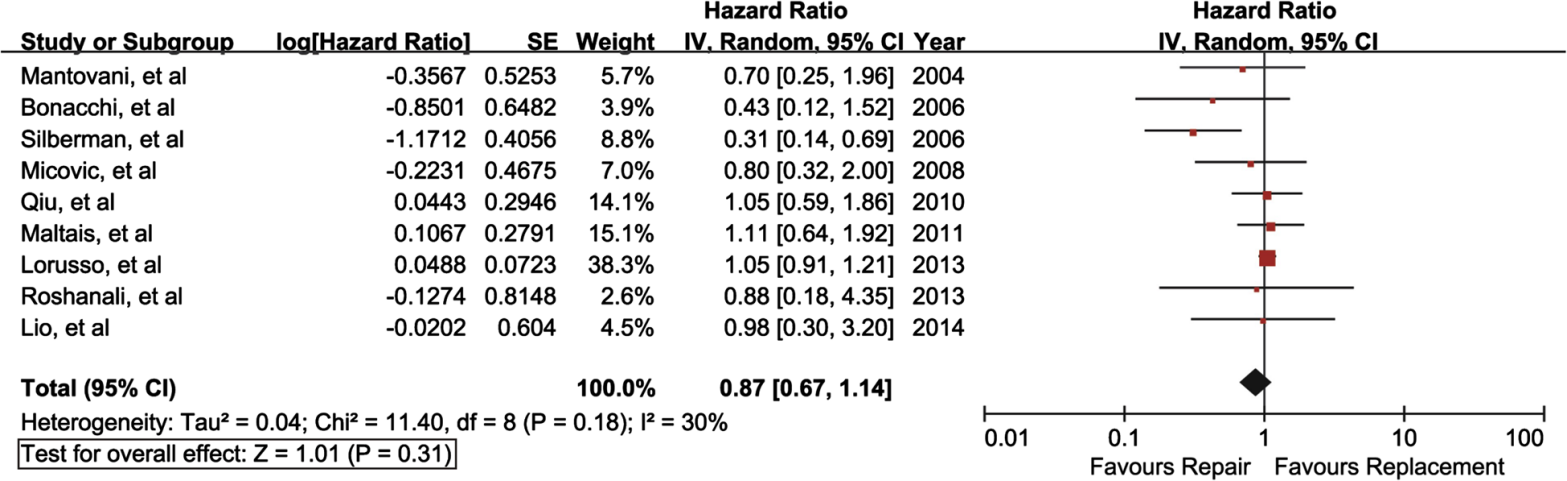
Mitral valve repair versus mitral valve replacement on late mortality

A total of nine studies (1622 Patients) reported late mortality (Fig. 3). The overall hazard ratio was 0.87 (95 % CI, 0.67-1.14; *P* = 0.31), suggesting late mortality was not significantly reduced following repair. Further, heterogeneity was moderate (I^2^ = 30 %). It was noted that ten of the studies included patients with different degrees of regurgitation and left ventricular dysfunction, with exception of one study [23], all of the patients included in this study had severely impaired LV function (ejection fraction <25 %) and severe ischemic MR undergoing CABG. Severely decreased left ventricular function and severe IMR could have the potential pathophysiological effect on the mortality rates of those patients. Hence, sensitivity analysis was conducted to only include studies in which not all of the patients had severe ischemic MR and severely impaired LV function undergoing CABG. Restricting analysis to these studies had no significant impact on the reduction of late mortality following repair (the summary hazard ratio, 1.03; 95 % CI, 0.90-1.17; *P* = 0.66). Whereas, heterogeneity suggested by I^2^ was significantly reduced to 0 %, indicating no variability exists among the rest studies. Further exclusion of any single study did not significantly reduce the heterogeneity. In addition, our study included 10 retrospective studies and 1 prospective study. The different study designs may influence outcomes of meta-analysis. Therefore, sensitivity analysis was performed to only include retrospective studies. Restricting analysis to these studies did not significantly impact on the result for late mortality (HR, 0.86; 95 % CI, 0.64-1.14; *P* = 0.30; I^2^ = 38 %).

### Mitral valve reoperation

**Fig. 4:**
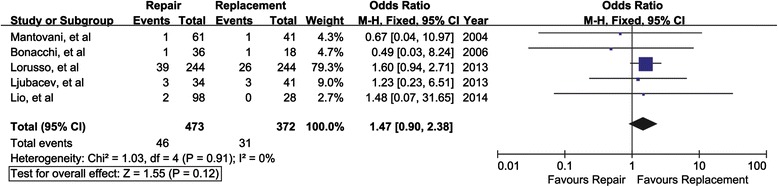
Mitral valve repair versus mitral valve replacement on reoperation

Reoperation due to such as MV regurgitation, thromboembolism and prosthetic endocarditis was reported in five studies involving a total of 845 patients. The combined odds ratio was 1.47, suggesting the trend went towards the preference of replacement. Nevertheless, no significant differences were reached between the two surgical approaches (95 % CI, 0.90-2.38; I^2^ = 0 %; *P* = 0.12) (Fig. 4).

### Recurrence of MR

**Fig. 5:**
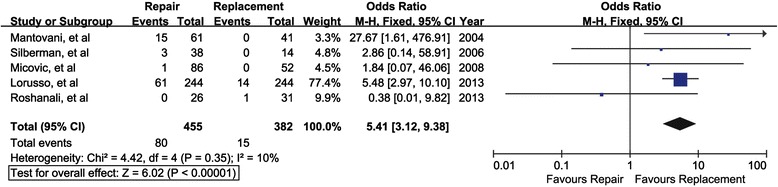
Mitral valve repair versus mitral valve replacement on recurrence of mitral valve regurgitation

Five studies involving a total of 837 patients provided data regarding recurrence of MR during the follow-up. The MVP + CABG group was associated with a significantly increased recurrence rate of MR (OR, 5.41; 95 % CI: 3.12–9.38; *P* < 0.001) with low heterogeneity among those studies (I^2^ = 10 %) (Fig. 5). Sensitivity analysis was also performed to only include retrospective studies. Restricting analysis to these studies did not significantly impact on the result for recurrence of MR (OR, 5.97; 95 % CI, 3.36-10.58; *P* < 0.001; I^2^ = 0 %).

## Discussion

In our meta-analysis of eleven studies, which included patients undergoing repair or replacement electively with CABG surgery, no differences were found regarding peri-operative mortality and long-term survival. Mitral valve replacement was associated with lower incidence of mitral regurgitation in patients with IMR during CABG. The Society of Thoracic Surgeons reports MVP + CABG group had approximately 5 % (4.8 % in-hospital mortality and 5.3 % operative mortality) nationwide mortality rates in contrast with 8 % (7.8 % in-hospital mortality and 8.5 % operative mortality) for MVR + CABG group [29].

Moderate and severe recurrent MR after a restrictive annuloplasty ring, occurred early and affected a substantial proportion of patients by 2 years [30]. In the present study, the main disadvantage of MVP + CABG group is recurrence of MR compared to MVR + CABG group. Although MR recurrence was common, mitral valve reoperation was not. Similar reoperation rate was found between both groups, which suggested that not all patients with MR recurrence 2+ or greater needed reoperation. There were several possible explanations. IMR after annuloplasty might be considered inconsequential compared with the underlying myocardial disease, or surgeons might be hesitant to perform a second or third cardiac operation in these elderly, high-risk patients [31]. Many patients with recurrent MR were just too sick or too old or both to even consider reoperating on them.

A case-matched study found that replacement was associated with lower incidence of valve-related complications than was repair and both mitral valve procedures showed no significant difference in LV function at follow-up [32]. However, replacement had greater thromboembolic and ischemic stroke rates than repair despite anticoagulant therapy [33]. Although mitral valve replacement can sufficiently correct regurgitation, the structural integrity of the mitral valve is usually compromised after replacement, leading to a continuous damage on the left ventricular tethered loop, which results in adverse effects on left ventricular contraction and poor prognosis [8]. Therefore, individualized consideration should be given to the two surgical procedures.

To date, there have been no RCTs that compared the clinical outcomes of the two surgical management particularly in patients with chronic IMR during CABG. To our knowledge, our report is the first meta-analysis comparing short-term and long-term outcomes of two mitral valve procedures specifically on patients with chronic IMR undergoing concomitant CABG. We selected studies for this meta-analysis with rigorous inclusion and exclusion criteria. All the patients in the studies underwent concomitant CABG, which ensures homogeneity of IMR patients and facilitates comparisons between trials. In addition, patients with acute IMR due to ruptured papillary muscles were excluded in our study, thus the outcomes of this meta-analysis not only truly reflect the surgical intervention of chronic IMR but also avoid biasing the results toward worsening the replacement group. By excluding articles that had > 20 % lack of annuloplasty ring, we have made the comparison between the two mitral valves surgeries more powerful. Therefore, the results of our study truly reflect the surgical management of patients with IMR simultaneous to CABG.

### Limitations

Our meta-analysis has several limitations. Firstly, this study was based on observational, retrospective studies with inherent bias of such study designs. The publications included in this meta-analysis were relatively small and nonrandomized studies. Secondly, changes in NYHA class, LVEF and left ventricular reversal remodeling were too scarcely reported in the included studies to enable meta-analysis. Eight out of eleven studies included in our meta-analysis reported data on the subvalvular apparatus preservation in mitral valve replacement yet with lack of uniform preservation of both the anterior and posterior leaflets. The other three studies had no description regarding subvalvular apparatus preservation. Thirdly, potential confounding factors such as preoperative risk evaluation (STS score i.e.), mitral valve more suitable for repair, age, cause of mitral regurgitation (ischemia, fibrosis, ventricular remodeling), EF and complexity of revascularization were not considered or adjusted in some of the studies included in our meta-analysis. Therefore, the superiority of repair over replacement may be affected by this and other factors that are not possible to be revealed with meta-analysis of observational trials. Well-designed RCTs are required to further verify the conclusion. Another limitation of our report is the fact that follow-up periods were heterogeneous between some studies with different use of mean and median durations of follow-up. Therefore, subgroup analysis could not be performed statistically.

## Conclusions

In patients with chronic ischemic mitral regurgitation during CABG, mitral valve replacement is associated with lower recurrence of regurgitation. No differences were found regarding survival and reoperation rates.

## References

[1] Piérard LA, Carabello BA (2010). Ischaemic mitral regurgitation: pathophysiology, outcomes and the conundrum of treatment. Eur Heart J.

[2] Lamas GA, Mitchell GF, Flaker GC, Smith SC, Gersh BJ, Basta L (1997). Clinical significance of mitral regurgitation after acute myocardial infarction. Circulation.

[3] Aklog L, Filsoufi F, Flores KQ, Chen RH, Cohn LH, Nathan NS (2001). Does coronary artery bypass grafting alone correct moderate ischemic mitral regurgitation?. Circulation.

[4] Lam BK, Gillinov AM, Blackstone EH, Rajeswaran J, Yuh B, Bhudia SK (2005). Importance of moderate ischemic mitral regurgitation. Ann Thorac Surg.

[5] Fattouch K, Guccione F, Sampognaro R, Panzarella G, Corrado E, Navarra E (2009). POINT: efficacy of adding mitral valve restrictive annuloplasty to coronary artery bypass grafting in patients withmoderate ischemic mitral valve regurgitation: a randomized trial. J Thorac Cardiovasc Surg.

[6] Smith PK, Puskas JD, Ascheim DD, Voisine P, Gelijns AC, Moskowitz AJ (2014). Cardiothoracic surgical trials network investigators. Surgical treatment of moderate ischemic mitral regurgitation. N Engl J Med.

[7] Dayan V, Soca G, Cura L, Mestres CA (2014). Similar survival after mitral valve replacement or repair for ischemic mitral regurgitation: a meta-analysis. Ann Thorac Surg.

[8] Wang J, Gu C, Gao M, Yu W, Yu Y (2015). Mitral valve replacement therapy causes higher 30-day postoperative mortality than mitral valvuloplasty in patients with severe ischemic mitral regurgitation: a meta-analysis of 12 studies. Int J Cardiol.

[9] Lorusso R, Gelsomino S, Vizzardi E, D’Aloia A, De Cicco G, Lucà F (2013). Mitral valve repair or replacement for ischemic mitral regurgitation? the Italian study on the treatment of ischemic mitral regurgitation (ISTIMIR). J Thorac Cardiovasc Surg.

[10] Lio A, Miceli A, Varone E, Canarutto D, Di Stefano G, Della Pina F (2014). Mitral valve repair versus replacement in patients with ischaemic mitral regurgitation and depressed ejectionfraction: risk factors for early and mid-term mortality. Interact Cardiovasc Thorac Surg.

[11] Micovic S, Milacic P, Otasevic P, Tasic N, Boskovic S, Nezic D (2008). Comparison of valve annuloplasty and replacement for ischemic mitral valve incompetence. Heart Surg Forum.

[12] Bonacchi M, Prifti E, Maiani M, Frati G, Nathan NS, Leacche M (2006). Mitral valve surgery simultaneous to coronary revascularization in patients with end-stage ischemiccardiomyopathy. Heart Vessels.

[13] Maltais S, Schaff HV, Daly RC, Suri RM, Dearani JA, Sundt TM (2011). Mitral regurgitation surgery in patients with ischemic cardiomyopathy and ischemic mitral regurgitation: factors that influence survival. J Thorac Cardiovasc Surg.

[14] Vahanian A, Alfieri O, Andreotti F, Antunes MJ, Barón-Esquivias G, Baumgartner H (2012). Guidelines on the management of valvular heart disease (version 2012). Eur Heart J.

[15] Nishimura RA, Otto CM, Bonow RO, Carabello BA, Erwin JP, Guyton RA (2014). 2014 AHA/ACC guideline for the management of patients with valvular heart disease: a report of the American College of Cardiology/American Heart Association Task Force on Practice Guidelines. J Am Coll Cardiol.

[16] Stroup DF, Berlin JA, Morton SC, Olkin I, Williamson GD, Rennie D (2000). Meta-analysis of observational studies in epidemiology: a proposal for reporting. Metaanalysis of observational studies in epidemiology (MOOSE) group. JAMA.

[17] Wells GA, Shea B, O’Connell D, Peterson J, Welch V, Losos M (2011). The Newcastle-Ottawa scale (NOS) for assessing the quality if nonrandomized studies in meta-analyses.

[18] Tierney JF, Stewart LA, Ghersi D, Burdett S, Sydes MR (2007). Practical methods for incorporating summary time-to-event data into meta-analysis. Trials.

[19] DerSimonian R, Laird N (1986). Meta-analysis in clinical trials. Control Clin Trials.

[20] Higgins JP, Thompson SG, Deeks JJ, Altman DG (2003). Measuring inconsistency in meta-analyses. BMJ.

[21] Egger M, Smith GD, Schneider M, Schneider M, Minder C (1997). Bias in meta-analysis detected by a simple, graphical test. BMJ.

[22] Begg CB, Mazumdar M (1994). Operating characteristics of a rank correlation test for publication bias. Biometrics.

[23] Silberman S, Oren A, Klutstein MW, Deeb M, Asher E, Merin O (2006). Does mitral valve intervention have an impact on late survival in ischemic cardiomyopathy?. Isr Med Assoc J.

[24] Ljubacev A, Medved I, Ostrik M, Zuvić-Butorac M, Sokolić J (2013). Mitral regurgitation and coronary artery bypass surgery: comparison of mitral valve repair and replacement. Acta Chir Belg.

[25] Mantovani V, Mariscalco G, Leva C, Blanzola C, Cattaneo P, Sala A (2004). Long-term results of the surgical treatment of chronic ischemic mitral regurgitation: comparison of repair andprosthetic replacement. J Heart Valve Dis.

[26] Qiu Z, Chen X, Xu M, Jiang Y, Xiao L, Liu L (2010). Is mitral valve repair superior to replacement for chronic ischemic mitral regurgitation with left ventricular dysfunction?. J Cardiothorac Surg.

[27] Reece TB, Tribble CG, Ellman PI, Maxey TS, Woodford RL, Dimeling GM (2004). Mitral repair is superior to replacement when associated with coronary artery disease. Ann Surg.

[28] Roshanali F, Vedadian A, Shoar S, Sandoughdaran S, Naderan M, Mandegar MH (2013). When to repair ischemic mitral valve regurgitation? an algorithmic approach. Eur Surg.

[29] Adult cardiac surgery database. Society of Thoracic Surgeons, Chicago. 2012. http://www.sts.org/national-database. Accessed 17 Dec 2015.

[30] Kron IL, Hung J, Overbey JR, Bouchard D, Gelijns AC, Moskowitz AJ (2015). Predicting recurrent mitral regurgitation after mitral valve repair for severe ischemic mitral regurgitation. J Thorac Cardiovasc Surg.

[31] McGee EC, Gillinov AM, Blackstone EH, Rajeswaran J, Cohen G, Najam F (2004). Recurrent mitral regurgitation after annuloplasty for functional ischemic mitral regurgitation. J Thorac Cardiovasc Surg.

[32] Chan V, Ruel M, Mesana TG (2011). Mitral valve replacement is a viable alternative to mitral repair for ischemic mitral regurgitation: a case-matched study. Ann Thorac Surg.

[33] Russo A, Grigioni F, Avierinos JF, Freeman WK, Suri R, Michelena H (2008). Thromboembolic complications after surgical correction of mitral regurgitation incidence, predictors, and clinical implications. J Am Coll Cardiol.

